# Integrated Phylogenomics and Expression Profiling of the *TRM* Gene Family in *Brassica napus* Reveals Their Role in Development and Stress Tolerance

**DOI:** 10.3390/plants14121858

**Published:** 2025-06-17

**Authors:** Yunlu Zhang, Ke Zhao, Ruisen Wang, Yang Zhu, Huiqi Zhang, Jingyi Zhang, Xiangtan Yao, Cheng Qin, Pengcheng Zhang

**Affiliations:** 1 College of Life and Environmental Sciences, Hangzhou Normal University, Hangzhou 311121, China; 2022111010011@stu.hznu.edu.cn (Y.Z.); 2022111010028@stu.hznu.edu.cn (K.Z.); 2 Institute of Economic Crop Sciences, Jiaxing Academy of Agricultural Sciences, Jiaxing 314016, China; wangruisen@hotmail.com (R.W.); yxt156@hotmail.com (X.Y.); 3 Institute of Crop Science, Zhejiang University, Hangzhou 310058, China; zhuyang2020@zju.edu.cn (Y.Z.); zhq2022@zju.edu.cn (H.Z.); zjy2023@zju.edu.cn (J.Z.)

**Keywords:** *TRM* (*TONNEAU1 Recruiting Motif*) gene, bioinformatics analysis, *Brassica napus*, abiotic stress

## Abstract

The *TRM* (*TONNEAU1 Recruiting Motif*) gene family plays a crucial role in multiple biological processes, including microtubule organization, cell division regulation, fruit morphogenesis, stress adaptation, and growth and development. To delve deeper into the potential functions of *BnaTRMs* in *Brassica napus*, this study employed bioinformatics methods to systematically identify and analyze the *TRM* family genes in *Brassica napus* (Westar). Using the model plant *Arabidopsis thaliana* as a reference and based on six conserved motifs, 100 *TRM* members were first identified in *Brassica napus*. These genes are widely distributed across 19 chromosomes, and most exhibit nuclear localization characteristics. Through gene collinearity analysis among *Brassica napus*, *Arabidopsis thaliana*, *Glycine max*, *Oryza sativa*, and *Zea mays*, we speculate that *Brassica napus* and *Glycine max* may share a similar evolutionary history. Analysis of cis-acting elements in the 2000 bp upstream region of *TRM* gene promoters revealed numerous elements related to abiotic stress response and hormone regulation. Furthermore, qRT-PCR data supported these findings, indicating that multiple *TRM* genes actively participate in the growth and development process and abiotic stress tolerance of *Brassica napus*. In summary, *BnaTRMs* exhibit significant functions in stress adaptation, growth, and development. This study not only enhances our understanding of the functions of the *TRM* gene family but also provides new perspectives and strategies for further exploring their regulatory mechanisms and potential applications.

## 1. Introduction

*Brassica napus* L. is one of the vital oilseed crops in China [1], the allotetraploid rapeseed (A_n_A_n_C_n_C_n_, 2n = 4x = 38) originates from spontaneous hybridization of its diploid ancestors *Brassica rapa* (A_r_A_r_, 2n = 2x = 20) and *Brassica oleracea* (C_o_C_o_, 2n = 2x = 18) [2,3,4]. In agricultural ecology, the root system of *Brassica napus* L. plays a crucial role in improving soil structure, enhancing soil fertility, and preventing water and soil loss. Additionally, planting *Brassica napus* L. can effectively suppress weed growth, thereby reducing pesticide usage and benefiting ecological conservation. The development of the *Brassica napus* L. industry is of great significance in ensuring national food security, promoting economic growth, and maintaining ecological balance.

The *TRM* gene family plays crucial roles in plant growth and development. In *Arabidopsis thaliana*, 34 TRM proteins have been identified, half of which are microtubule-associated proteins involved in microtubule organization formation and maintenance [5,6]. Charge analysis of *TRM* primary sequences reveals that approximately half of *TRM* family members contain large basic regions, suggesting these proteins likely participate in microtubule binding [7,8]. Microtubules serve as essential structural elements within the plant cytoskeleton, fulfilling critical functions in cellular morphology maintenance and adaptive responses during growth, developmental processes, and environmental fluctuations. Additionally, they participate in fundamental cellular activities, including mitotic division, organelle trafficking, pathogen defense mechanisms, and stress adaptation [9,10]. TONNEAU1 (TON1) protein represents a highly conserved acidic protein in land plants [11]. TONNEAU1 Recruiting Motif (TRM) proteins derive their nomenclature from their functional association with TON1, a plant-specific protein homologous to the human centrosomal FOP protein that is critically required for proper microtubule array formation in *Arabidopsis thaliana* [5]. The TON1 proteins serve as a crucial regulator of leaf and silique morphology in *Arabidopsis thaliana* [12], grain shape in *Oryza sativa* L. [13,14], as well as fruit morphology in *Solanum lycopersicum* and *Cucumis sativus* L. [15]. The TRM proteins interact with TON1 and protein phosphatase 2A (PP2A) through their M2 and M3 domains, respectively, forming the TON1-TRM-PP2A (TTP) protein complex. This complex specifically localizes to microtubules (MTs), where it regulates microtubule organization and preprophase band (PPB) formation, thereby modulating cell division and growth processes that ultimately determine the size and morphology of plant organs [6,16,17,18,19,20].

In *Arabidopsis thaliana*, the *Attrm5* mutant exhibits retarded leaf growth, delayed flowering, and reduced root length [21]. *AtTRM61* possesses conserved functional domains, including a characteristic motif for S-adenosyl-L-methionine (AdoMet) cofactor binding, which plays a crucial role in regulating embryonic arrest and seed abortion processes [22]. The research demonstrates that *AtTRM1* and *AtTRM2* regulate leaf morphology by actively promoting longitudinal polar cell elongation [5]. These two genes play crucial roles in determining final leaf shape and size through modulating cell elongation patterns. This regulatory mechanism reveals the precise control function of *TRM* family genes in plant organ morphogenesis, providing important clues for understanding the molecular mechanisms of plant organ development. Recent studies show that *AtTRM21* positively regulates flavonoid biosynthesis at the translational level in *Arabidopsis thaliana*. Loss-of-function mutation in *TRM21* leads to root hair growth defects and delayed plant growth, accompanied by significant alterations in secondary metabolites, particularly a marked reduction in flavonoid content. This discovery not only reveals a novel metabolic regulatory function of *TRM21* but also provides new molecular targets for elucidating the regulatory network of flavonoid biosynthesis [23]. In rice, *TRM* homologous gene *OsGW7/GL7/SLG7* ultimately determines grain size and quality traits by regulating cell length and width [24,25]. In *Solanum lycopersicum*, TRM protein family members interact with the microtubule organizing center OFP to regulate fruit morphology by controlling cell division orientation and cell expansion. OVATE and SlOFP20 interact with SlTRM5 and ten other *TRM* family members. The TRM-OFP protein complexes exhibit dynamic subcellular localization in the cytoplasm and microtubules, dependent on their co-expression patterns, suggesting that their spatial distribution may contribute to morphological regulation [26,27,28,29]. The research demonstrates that this TRM-OFP interaction network precisely coordinates cellular behaviors during fruit development, ultimately determining final fruit shape. In *Cucumis sativus* L., some *CsTRM* genes are induced or suppressed at different time points under stress treatments. Particularly, *CsTRM21* shows significant expression variations between long and short fruited cultivars, as well as under abiotic stresses (salt and heat) and biotic stresses (powdery mildew and gray mold), suggesting its dual role in determining fruit shape and stress resistance [30].

As a crucial oilseed crop, rapeseed (*Brassica napus* L.) is particularly susceptible to abiotic stresses, including chilling stress, drought stress, and salt stress, which significantly impair its growth dynamics and developmental processes and ultimately compromise both yield potential and oil quality traits [31]. Under abiotic stress conditions, the dynamic reorganization of the microtubule cytoskeleton plays a critical role in maintaining cellular homeostasis [32]. These three abiotic stresses can significantly disrupt the structure and function of microtubules in plant cells, thereby interfering with critical physiological processes, including cell division, cell expansion, cell morphogenesis, and intracellular transport. Under cold stress conditions, low temperatures destabilize the polymerization equilibrium of microtubule subunits, leading to aberrant cell division patterns that ultimately manifest as phenotypic abnormalities such as leaf wrinkling and organ deformation [33]. Under drought stress conditions, microtubules mediate stomatal closure, consequently reducing transpiration rates and compromising cellular osmoregulatory capacity (microtubule-associated protein AtMPB2C plays a role in the organization of cortical microtubules, stomata patterning, and tobamovirus infectivity). Under high-salinity stress conditions, cortical microtubules in plant cells undergo depolymerization, disrupting microtubule dynamic equilibrium and consequently impairing tissue development and organ morphogenesis [34]. Notably, these stresses often exert synergistic effects on the microtubule system. For instance, the combined action of salinity–alkalinity and drought stress significantly exacerbates microtubule network disorganization, while low-temperature stress further amplifies these disruptive effects. Members of the *TRM* gene family serve as crucial regulators of plant growth and stress responses, playing pivotal roles in plant morphogenesis and stress adaptation through their involvement in microtubule organization and cell division regulation. The evolutionary conservation of this gene family across diverse plant species makes it an important target for crop genetic improvement. However, to date, there have been no reported studies investigating the participation of *BnaTRM* family genes in abiotic stress responses in rapeseed.

In this study, we systematically identified 100 *BnaTRM* genes with high homology to *Arabidopsis thaliana* in the *Brassica napus* Westar cultivar using bioinformatics approaches. Comprehensive analyses were conducted to characterize their structural features and chromosomal distributions, and a multi-species phylogenetic tree was constructed, including *Arabidopsis thaliana*, *Glycine max*, *Oryza sativa*, and *Zea mays*. Furthermore, quantitative expression profiling revealed stress-specific expression patterns among *BnaTRM* family members, with several key genes exhibiting distinctive regulatory profiles that suggest functional specialization in response to multiple stresses. Our findings not only fill a critical knowledge gap in rapeseed *TRM* gene family research but also establish a foundation for investigating the potential roles of *TRM* in stress tolerance mechanisms in *Brassica napus*.

## 2. Results

### 2.1. One Hundred TON1 Recruiting Motif Family Members in Westar Were Mainly Divided into Eight Subfamilies

**Figure 1:**
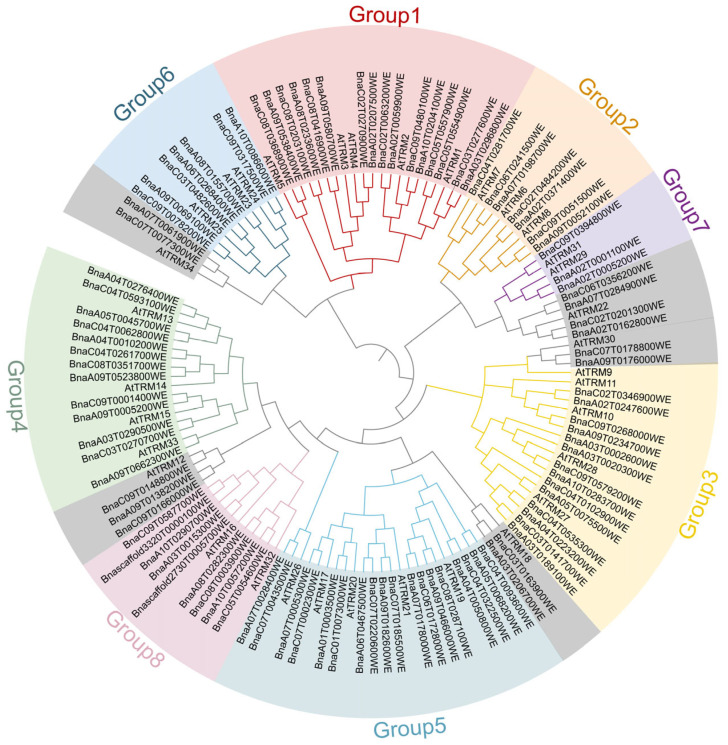
Phylogenetic analysis of the *TRM* family genes of *Brassica napus* and *Arabidopsis thaliana*. The protein sequence alignments and construction of the phylogenetic tree were performed using MEGA 11.0.13 and the neighbor-joining method with 1000 bootstrap replicates. The different colors represent eight subfamilies of the *TRM* gene family; branches indicate different evolutionary clades. Gray represents independent branches.

The *TON1 Recruiting Motif* (*TRM*) has been identified in *Arabidopsis thaliana*, a cruciferous plant. Consequently, using the amino acid sequences of *AtTRMs* as the source, a Blast search was conducted against the *Brassica napus* genome database, yielding 128 genes with E-values less than 1.0 × 10^−5^. Using the MEME v5.5.7 database to analyze their motifs, those with the conserved motifs were designated members of the *TRM* gene family. Thus, 100 *TRM* family members were identified (Table 1). To further understand the evolutionary relationships among the *TRM* family members in *Brassica napus*, comparative sequence alignments of *TRM* genes were conducted for *Brassica napus* and *Arabidopsis thaliana*, with subsequent generation of an unrooted phylogenetic tree utilizing MEGA 11.0.13 software. As shown in Figure 1, the rapeseed *TRM* family is divided into eight subfamilies and some independent branches. The largest clade, Group 3, comprises 18 BnaTRM members and five AtTRM proteins. In contrast, the smallest clade, Group 7, contains three BnaTRM and two AtTRM proteins (Figure 1).

The remarkable expansion of the *TRM* gene family in *Brassica napus* (containing 100 members) may be intrinsically linked to whole-genome duplication events during polyploidization. The retention of 652 homologous gene pairs between the A/C subgenomes likely facilitated functional module differentiation [35,36] (e.g., stress response-related subfamilies) and redundancy buffering mechanisms, thereby synergistically reinforcing adaptive evolutionary advantages in response to complex environmental stresses.

### 2.2. Gene Motif and Structure of the BnaTRMs

**Figure 2:**
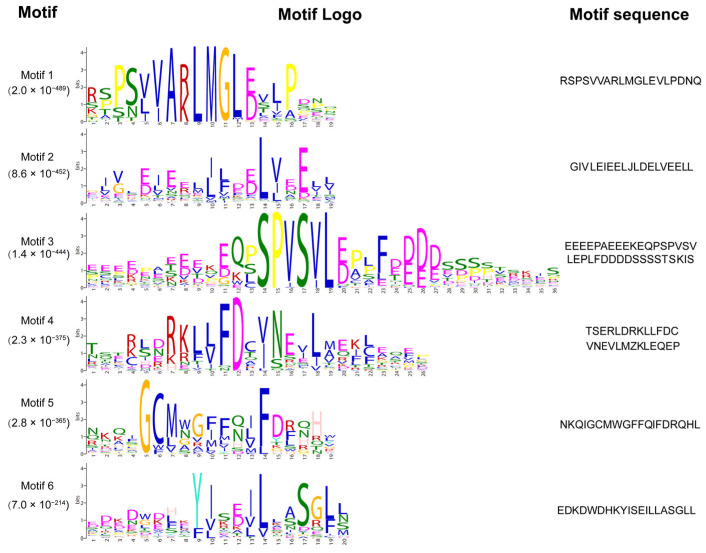
Sequences of top six enriched motifs identified by MEME v5.5.7. The MEME v5.5.7 tool was used with the following parameters: protein; nostatus; mod anr; nmotifs 6; minsites 17; minw 10; maxw 100.

**Figure 3:**
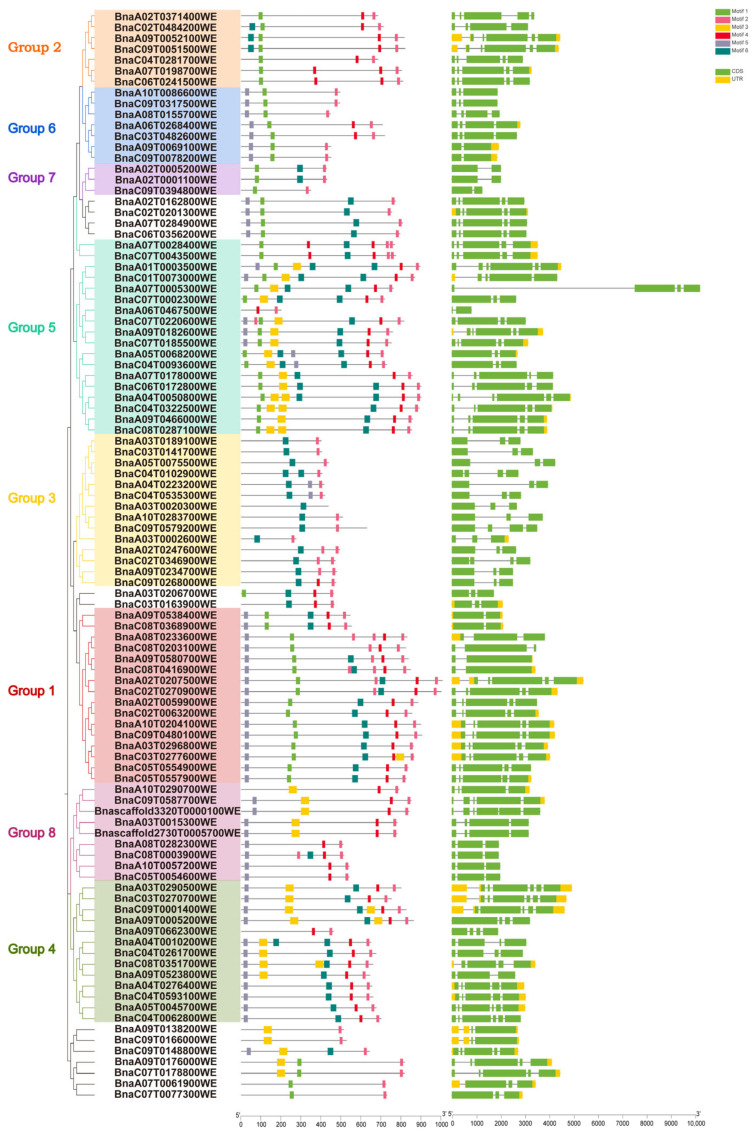
Sequence logo representation of top eight enriched six motifs defined from the 100 BnaTRM proteins by MEME v5.5.7.

In *Arabidopsis thaliana*, 34 TRM proteins possess six highly conserved motifs, which are strictly conserved in the order of M5-M1-M3-M6-M4-M2 along the protein sequence. All 34 AtTRMs contain motif M2 at their C-termini, potentially serving as a characteristic feature of the entire *TRM* superfamily. Based on this feature, 100 *BnaTRM* genes conforming to this characteristic were selected from the 128 homologous genes, and their motifs were subjected to visual analysis (Figure 2). Some *BnaTRM* only possess two conserved motifs, while most of them own 4–5 (Figure 3). The M1-M6 motif in *Brassica napus* is slightly longer than the M1-M6 motif in *Arabidopsis thaliana*, but the sequence is relatively conserved. The conservation of the M2 motif ensures the stability of core functions such as microtubule nucleation [11], whereas the dynamic loss of variable motifs (e.g., M4) constitutes an evolutionary trade-off mechanism. This evolutionary strategy not only preserves the functional integrity of TRM proteins but also endows plants with environmental adaptability innovations through module replacement. This evolutionary paradigm exhibits significant convergence with the module recombination mechanisms observed in the *TRM* gene family of *Cucumis sativus* L. [30].

To further investigate the gene structure, a comparison was conducted between the CDS and genomic sequences of the *BnaTRM* genes. The comparison results showed that *BnaA02T0005200WE*, *BnaA02T0001100WE*, *BnaC09T0394800WE*, *BnaA06T0467500WE*, *BnaA09T0538400WE*, *BnaC08T0368900WE*, *BnaA09T0580700WE*, and *BnaC08T0416900WE* comprised only two exons. The remaining genes contained at least three exons (Figure 3). The difference in the number of exons reflects the hierarchical complexity of gene expression regulation: double-exon genes (such as *BnaA02T0005200WE*) may achieve tissue-specific expression through cis-elements in the promoter region (such as ABRE/ARE), while multi-exon genes are more likely to produce functional isomers through alternative splicing to meet the needs of different development stages.

### 2.3. Chromosome Distributions of the BnaTRM Genes

**Figure 4:**
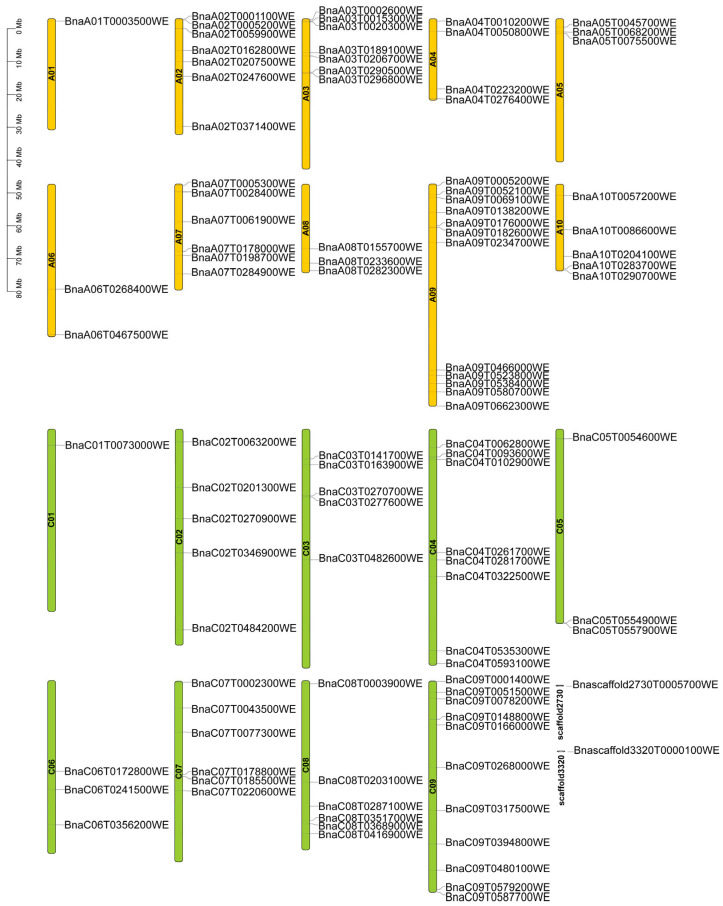
Chromosome locations of *BnaTRM* family genes. The length of the bars indicates the sizes of *Brassica napus* chromosomes. The physical locations of the genes are labeled on the left of the chromosomes, and the genes are labeled on the right of the chromosomes.

To gain a more intuitive understanding of the distribution of *TRM* genes across the chromosomes of *Brassica napus*, we utilized TBtools v2.310 to create a chromosomal distribution map (Figure 4). Among the 19 chromosomes of Westar, 98 *BnaTRM* genes are distributed. Specifically, 50 *BnaTRMs* are located in the A subgenome, and 48 *BnaTRMs* are located in the C subgenome. Two genes, namely *Bnascaffold2730T0005700WE* and *Bnascaffold3320T0000100WE*, could not be mapped to specific chromosomes in the Westar genome and are instead situated on scaffolds2730, respectively. Chromosomes A09 and C09 contain the highest number of *BnaTRM* genes, each containing 11, while chromosomes A01 and C01 each contain only one *BnaTRM* gene. It is suggested that A09 and C09 may be the core regulatory regions mediated by TRM. This distribution pattern reveals the adaptive evolutionary strategies of the *TRM* gene family following polyploidization in *Brassica napus*.

### 2.4. Synteny Analysis of BnaTRM Genes

**Figure 5:**
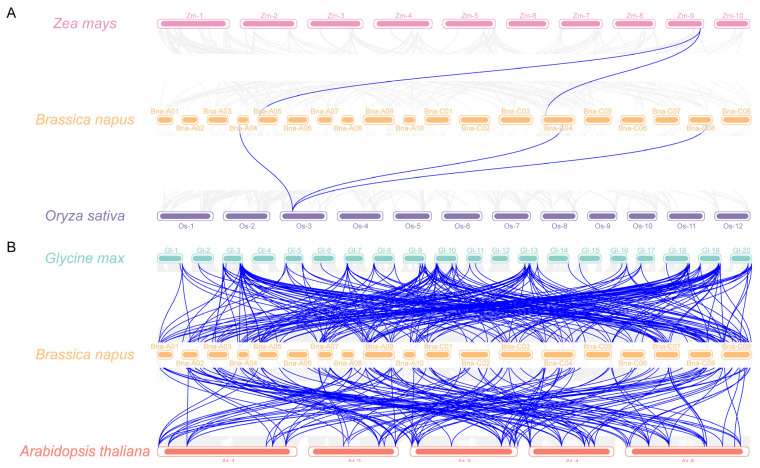
Analysis of syntenic relationships among *Oryza sativa*, *Zea mays* L. (**A**), *Arabidopsis thaliana*, and *Glycine max* (**B**). The gray line represents the syntenic block in plant genomes, and the blue line represents the collinear *TRM* gene pair.

To delve deeper into the phylogenetic relationships of the *Brassica napus TRM* family, we constructed a gene collinearity map for *Brassica napus* and compared it with four representative species: two monocotyledonous plants (*Oryza sativa* L. and *Zea mays*, Figure 5A) and two dicotyledonous plants (*Arabidopsis thaliana* and *Glycine max*, Figure 5B). The results indicated that among *Brassica napus* and these four species, only 2 pairs of *TRM* collinear gene pairs were identified between *Brassica napus* and *Zea mays*, followed by 3 pairs between *Brassica napus* and *Oryza sativa*, 152 pairs between *Brassica napus* and *Arabidopsis thaliana*, and 224 pairs between *Brassica napus* and *Glycine max*. Notably, the number of orthologous genes shared between dicotyledonous plants was significantly higher than that shared between dicotyledonous and monocotyledonous plants. This observation aligns with the expected pattern of biological evolution. The collinear gene pairs identified between *Brassica napus* and *Oryza sativa*, *Zea mays*, and *Arabidopsis thaliana* were also present in *Brassica napus* and *Glycine max*, suggesting a possible shared evolutionary history between *Brassica napus* and *Glycine max*.

### 2.5. The Biophysical Properties of BnaTRMs

The molecular weights of the BnaTRM proteins ranged from 22.545 kDa (*BnaA06T0467500WE*) to 111.547 kDa (*BnaA02T0207500WE*) in *Brassica napus*. The pI ranged from 4.37 (*BnaA03T0002600WE*) to 9.86 (*BnaC09T0078200WE*). On the basis of their pI, most BnaTRM proteins were acidic. Fifty-eight proteins are acidic, and thirty-seven proteins are alkaline. However, all of the BnaTRM proteins were unstable, with an instability index greater than 40. The Grand Average of the Hydropathicity index for all TRM proteins (<0) reflected the hydrophilic nature of these proteins. The aliphatic index for all BnaTRM proteins ranged from 59.27 (*BnaA05T0075500WE*) to 80.14 (*BnaA09T0538400WE*). The subcellular localization analysis revealed that the BnaTRM proteins were mostly localized in the nucleus (87 *BnaTRMs*), followed by the chloroplast (6 *BnaTRMs*) and cytoplasm (2 *BnaTRMs*) (Table 1). This analysis reveals the evolutionary strategy of the *BnaTRM* family to realize functional specialization through physical and chemical property differentiation.

### 2.6. Cis-Regulatory Elements Identification in Promoters

**Figure 6:**
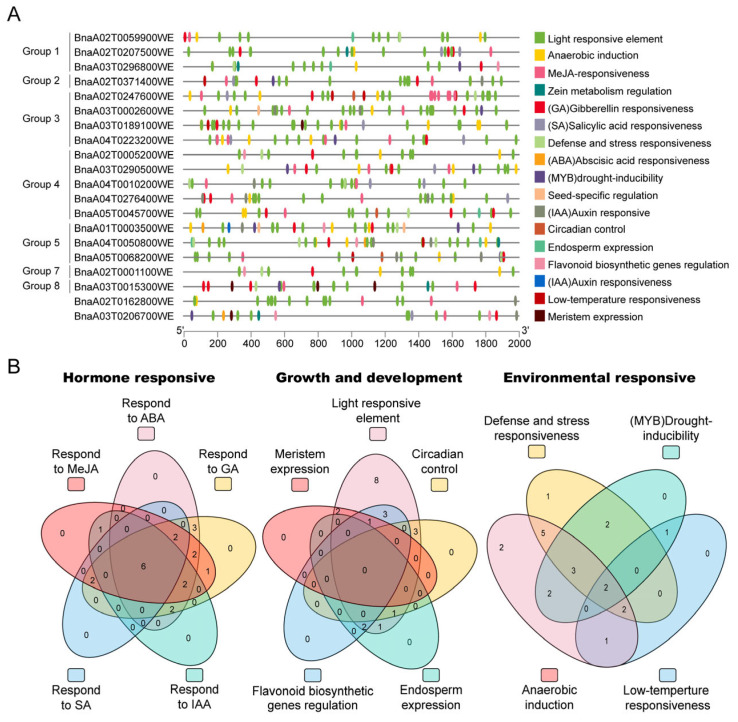
Cis-elements analysis in the promoter sequences of *BnaTRM* genes: (**A**) Visualization of the cis components of the *TRM* family with TBtools v2.310. (**B**) The Venn diagram is used to show the cis components under different paths.

Cis-acting elements in the promoter region are very important for regulating the expression of corresponding genes because they bind to various transcription factors. Thus, we identified the cis-regulatory elements in 2000 bp sequences upstream of the start codon of genes in *BnaTRM* using the PlantCARE database. As predicted, common promoter elements (TATA-box and CAAT-box) were identified among all *BnaTRM* gene promoters. Among 526 cis-acting elements, 233 cis-elements related to light, 172 cis-elements related to hormones, and 121 cis-elements with other functions were detected. Among the cis-acting elements related to hormones, 68, 42, 31, 18, and 13 are involved in MeJA, ABA, GA, IAA, and SA responsiveness. Therefore, *BnaTRMs* have the potential to play a role in a variety of processes. Forty-six light-responsive cis-elements involved in growth and development were detected in the *TRM* gene promoter regions (Table 2). Among the cis-acting elements involved in hormone responses, ABRE, GAREs (GARE-motif and P-box), and the MeJA-responsive elements (CGTCA-motif and TGACG-motif) were, respectively, identified in the promoter regions of 17, 19, and 16 *TRM* genes in *Brassica napus*. The zein metabolism regulation element (O2 site) was detected in six *BnaTRM* genes, whereas the CAT-box influencing meristem expression was identified in the promoter regions of three *BnaTRM* genes. Of the stress-related response elements, ARE, which is an anaerobic induction element, was detected in the promoters of 17 *TRM* genes. Some stress-related (low temperatures, wounding, and drought) cis-acting elements were also identified in the promoter regions of *TRM* genes. We use TBtools v2.310 to visualize these data, as shown in Figure 6. This analysis reveals the evolutionary strategy of the *BnaTRM* gene family to realize multi-dimensional environmental response through a modular combination of cis-acting elements, and the related functions of *BnaTRM* with core regulatory elements (such as ABRE and GAREs) can be verified first in the future.

### 2.7. Expression Profiling Analysis of 29 BnaTRM Genes Across 12 Distinct Tissues

**Figure 7:**
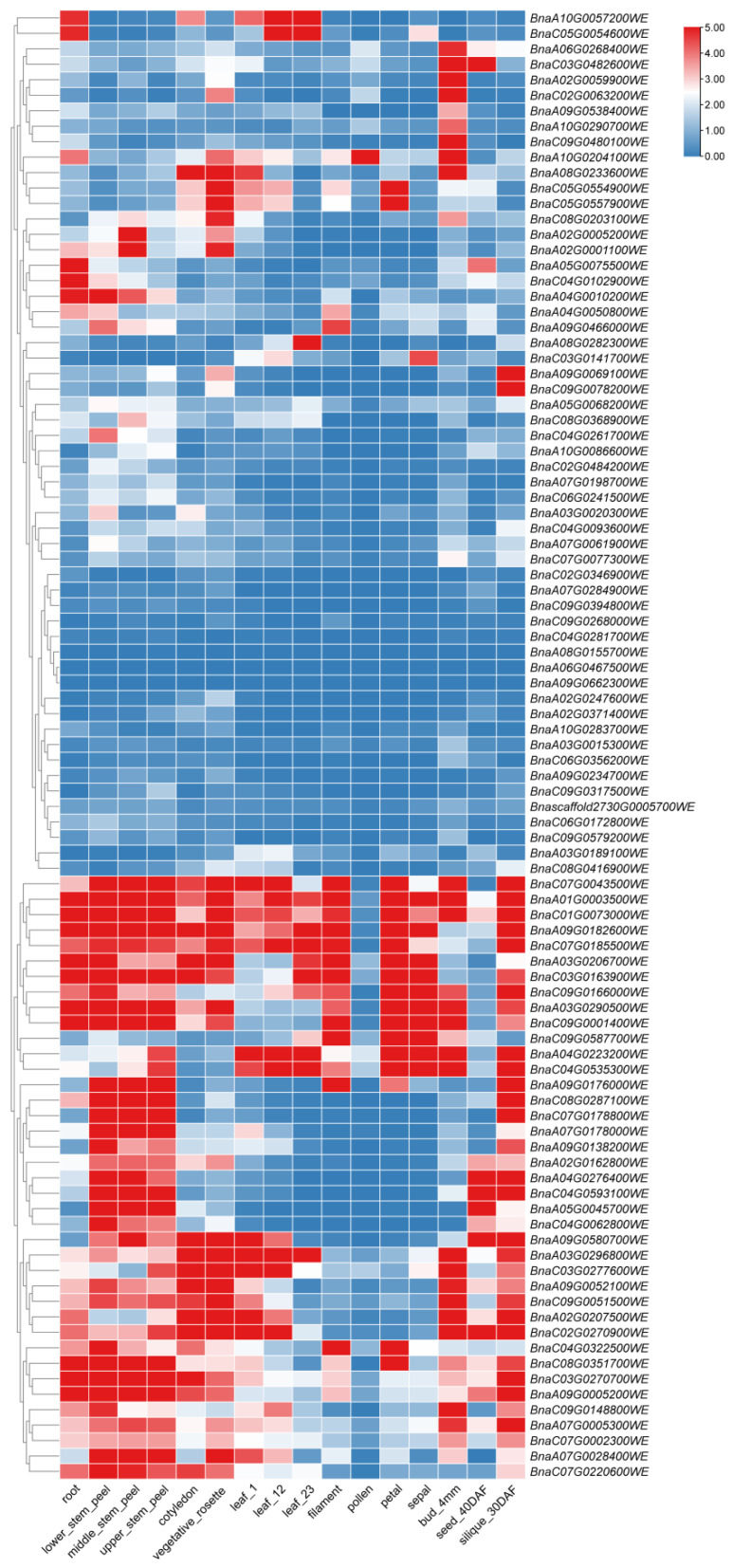
Expression patterns of *TRM* family genes in twelve tissues. The heat map was generated using log_2_ expression levels (TPM + 1). The bar indicates the log_2_ expression levels (TPM + 1). The legend label is shown on the right side of the figure. Genes with high expression levels are shown in red, and genes with ground expression levels are shown in blue. No dates are available for the *BnaA03T0002600WE*, *BnaA09T0523800WE*, *BnaC02T0201300WE*, *BnaC08T0003900WE*, and *Bnascaffold3320T0000100WE* genes. The RNA-seq data comes from the website BNIR (BnIR, *Brassica napus* multi-omics database (information resource).

Gene expression levels are typically closely associated with their biological functions. To investigate the functional roles of the *BnaTRM* genes family in *Brassica napus*, this study utilized the publicly available transcriptome database of the *Brassica napus* cultivar Zhongshuang 11 (ZS11) (https://yanglab.hzau.edu.cn/BnIR/expression_zs11 (accessed on 5 March 2024)). The *BnaTRM* genes family members from the Westar cultivar were homologously mapped to the ZS11 cultivar, and the expression patterns of *BnaTRM* genes across 12 different tissues (root, stem, cotyledon, vegetative rosette, cauline leaf, filament, pollen, petal, sepal, bud, seed, and silique) were analyzed using TBtools v2.310 software (Figure 7). The key findings revealed that most *BnaTRM* genes were upregulated in roots and stems. Five genes belonging to the Group 5 subfamily (*BnaC07T0043500WE*, *BnaA01T0003500WE*, *BnaC01T0073000WE*, *BnaA09T0182600WE*, *BnaC07T0185500WE*) exhibited low expression levels in pollen and seeds but showed high expression in the other 10 tissues. Eight genes from the Group 3 subfamily (*BnaC02T0346900WE*, *BnaC09T0268000WE*, *BnaA02T0247600WE*, *BnaA03T0189100WE*, *BnaA09T0234700WE*, *BnaA10T0283700WE*, *BnaC09T0579200WE*, *BnaA03T0020300WE*) displayed low expression across all tissues. These genes may regulate critical pathways in seed development (e.g., lipid biosynthesis or dormancy regulation), warranting further investigation into their association with agronomic traits such as seed size and oil content. The tissue-specific expression patterns of *BnaTRM* genes suggest that they are implicated in multiple regulatory processes during plant growth and development.

### 2.8. Differential Expression Analysis of BnaTRM Gene Family in Cold-Tolerant Varieties and Cold-Sensitive Varieties

**Figure 8:**
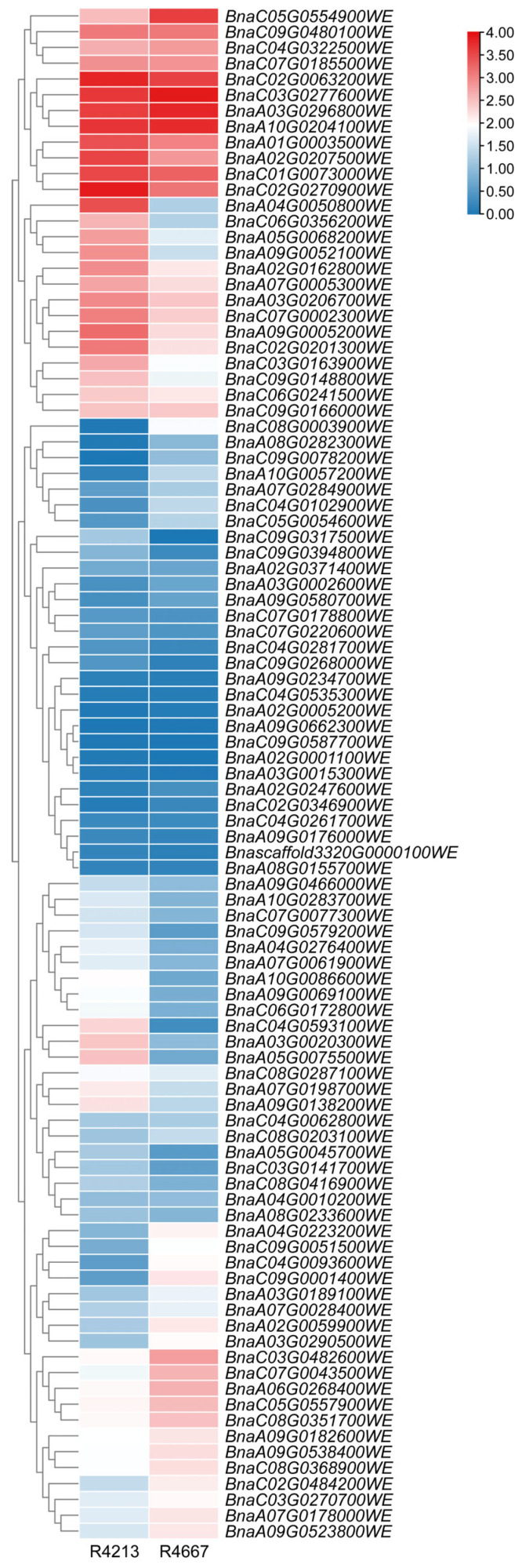
Expression patterns of *TRM* family genes in main inflorescence in cold-tolerant and cold-sensitive cultivars grown in the field. The heat map was generated using log_2_ expression levels (TPM + 1). The bars indicate the log_2_ expression levels (TPM + 1). The color scales represent relative expression levels from high (red color) to low (blue color). No dates are available for the *Bnascaffold2730G0005700WE*, *BnaA06G0467500WE*, and *BnaC04G0261700WE* genes.

Based on the method of cold-tolerant field-grown cultivar identification [37,38], we used the inflorescence materials of one cold-tolerant R4213 and another cold-sensitive R4667 cultivar for RNA-seq profiling. The heat map demonstrated significant divergence in *BnaTRM* expression profiles between the two groups (Figure 8). In cold-tolerant varieties, 31 genes such as *BnaA02T0207500WE*, *BnaA05T0068200WE*, and *BnaA02T0162800WE* showed remarkable upregulated patterns with extremely significant differential expression compared to the cold-sensitive varieties. These genes play a positive role in regulating cold stress. On the contrary, the expression levels of 23 genes, such as *BnaA02T0059900WE*, *BnaC05T0557900WE*, and *BnaA10T0057200WE*, in cold-tolerant varieties were significantly lower than those in cold-sensitive varieties, which may play a negative regulatory role in cold resistance. More than half of *BnaTRM* genes may respond to cold stress, indicating that *BnaTRM* genes may contribute to the low-temperature adaptation of *Brassica napus*.

### 2.9. Transcriptional Response of TRM Genes to Abiotic Stress in Brassica napus

**Figure 9:**
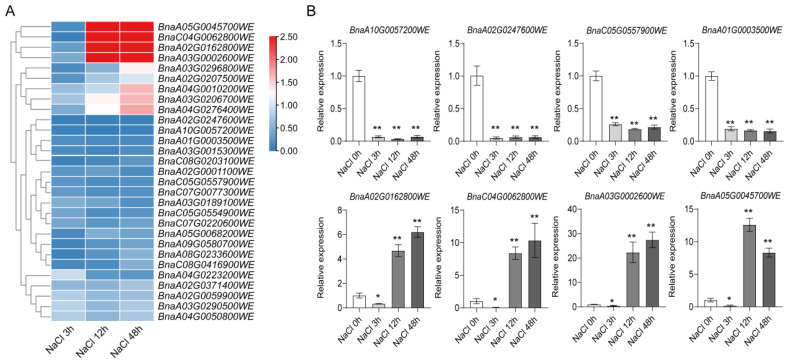
Expression level of *TRM* family genes after salt (NaCl) treatment at different times. (**A**) The heat map was generated using log_2_ expression levels (TPM + 1). The bars indicate the log_2_ expression levels (TPM + 1). The color scales represent relative expression levels from high (red color) to low (blue color). (**B**) The relative expression analysis of the *BnaTRMs* under salt (NaCl) treatment. The x-axis presents the treatments. The y-axis presents the expression levels relative to the expression at the 0 h time point. The data are representative of three independent experiments (n = 3, mean ± SD, * *p* < 0.05, ** *p* < 0.01, Student’s *t*-test).

**Figure 10:**
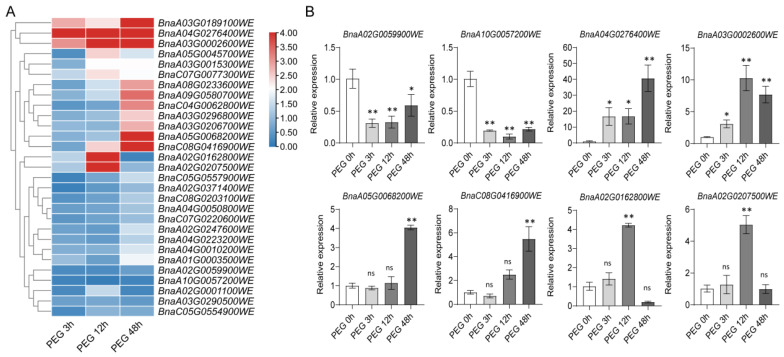
Expression level of *TRM* family genes after drought (PEG) treatment at different times: (**A**) The heat map was generated using log_2_ expression levels (TPM + 1). The bars indicate the log_2_ expression levels (TPM + 1). The color scales represent relative expression levels from high (red color) to low (blue color). (**B**) The relative expression analysis of the *BnaTRMs* under drought (PEG) treatment. The x-axis presents the treatments. The y-axis presents the expression levels relative to the expression at the 0 h time point. The data are representative of three independent experiments (n = 3, mean ± SD, * *p* < 0.05, ** *p* < 0.01, Student’s *t*-test) and ns represents no significant difference.

Abiotic stresses significantly impact plant growth and development. To investigate the effects of drought and salt stress on *TRM* gene expression in *Brassica napus*, we analyzed the transcriptional responses of 29 *TRM* genes under stress conditions using qRT-PCR. These 29 *TRM* genes include 20 genes containing cis-element, and 9 genes are homologous to the genes that are known to be involved in stress [30] (Figure 9A and Figure 10A). Under salt treatment, all *TRM* genes showed downregulated expression at 3 h post-treatment. Notably, four genes (*BnaA10T0057200WE*, *BnaA02T0247600WE*, *BnaC05T0557900WE*, and *BnaA01T0003500WE*) exhibited significantly reduced expression compared to controls. Interestingly, eight genes (*BnaA05T0045700WE*, *BnaC04T0062800WE*, *BnaA02T0162800WE*, *BnaA03T0002600WE*, *BnaA03T0206700WE*, *BnaA04T0276400WE*, *BnaA03T0296800WE*, and *BnaA04T0010200WE*) displayed unique dynamic regulation patterns, with significant upregulation observed at 12 h post-salt treatment (Figure 9B).

Meanwhile, after 3 h of drought treatment, we observed distinct transcriptional responses among *TRM* genes: *BnaA02T0059900WE* and *BnaA10T0057200WE* exhibited significant downregulation, while *BnaA04T0276400WE* and *BnaA03T0002600WE* showed remarkable upregulation with extremely significant differential expression (fold change ≥ 10) compared to controls. Notably, *BnaA05T0068200WE* and *BnaC08T0416900WE* displayed delayed response patterns, with significant upregulation only observed after 48 h of drought treatment. In contrast, *BnaA02T0162800WE* and *BnaA02T0207500WE* demonstrated biphasic regulation, showing upregulated expression at 12 h post-treatment, but they underwent significant downregulation by 48 h (Figure 10B). It is worth noting that *BnaA03T0002600WE* showed an upregulation under both drought and salt stress. It was demonstrated that this gene was positively involved in the stress response. However, *BnaA10T0057200WE* showed a continuous decrease under salt stress and drought stress, which may reflect its negative regulatory role in response to stress.

## 3. Discussion

TRMs are the TON1 Recruiting Motif (TRM) proteins, which share six short conserved motifs, including a TON1-interacting motif present in all TRMs. Members of the TRM protein family are generally positioned on microtubules and play a pivotal role in the establishment and preservation of microtubule architecture. Microtubules constitute a fundamental element of the plant cytoskeleton, essential for sustaining cellular shape, accommodating growth and development, and responding to environmental fluctuations. Furthermore, they are integral to processes such as cell division, material transportation, immune responses, and stress tolerance. Furthermore, it also contributes to regulating resistance to drought stress and plays an important role in plant growth and development.

In this study, we identified 100 *TRM* genes in *Brassica napus*, which is much higher than other species such as *Oryza sativa*, *Glycine max*, *Triticum aestivum*, and *Cucumis sativus*, perhaps because of complex multiple genome-wide duplication events in *Brassica napus* [39,40,41,42]. We used bioinformatics technology to conduct phylogenetic, collinearity, homology, gene structure, motif, chromosome location, expression, and cis-regulatory elements in promoter regions analyses for the *TRM* gene family members of *Brassica napus*. From this comprehensive gene family investigation, we drew three conclusions.

### 3.1. Genomic Architecture and Evolutionary Dynamics of TRM Genes in Brassica napus

The identification of 100 *BnaTRM* genes in *Brassica napus* reveals a complex genomic landscape shaped by evolutionary forces. The uneven distribution of *BnaTRM* genes across 19 chromosomes (with chromosomes A09 and C09 harboring 11 genes each) suggests functional clustering or tandem duplication events. This clustering may reflect the coordinated regulation of microtubule dynamics during specific developmental stages or stress responses. Comparative genomic analysis highlights the close evolutionary relationship between *Brassica napus* and *Glycine max*, with 224 collinear gene pairs identified. This synteny aligns with their shared *Brassicaceae* ancestry and supports the hypothesis of a common *TRM* gene family expansion in the *Brassiceae* tribe. In contrast, fewer orthologs were detected in monocots (*Oryza sativa* L. and *Zea mays*), indicating divergent evolutionary trajectories after the divergence of eudicots and monocots. Phylogenetic clustering into eight subfamilies (Groups 1–8) further underscores functional diversification, with Group 3 (18 genes) emerging as the largest clade.

### 3.2. Functional Prediction of BnaTRM Gene Family

Through comprehensive integrative analysis of the *BnaTRM* gene family (including cis-regulatory element identification, subcellular localization prediction, expression profiling, and quantitative validation), we systematically elucidated the multi-dimensional regulatory mechanisms of this gene family in plant stress responses. Promoter analysis revealed that *BnaTRM* genes are enriched with various stress-responsive elements (ARE, DRE, LTR) and hormone-responsive motifs (ABRE, MeJA-responsive CGTCA motif). Among them, 42 members harbor ABRE elements, suggesting their involvement in low-temperature, drought, and salt stress response pathways; 16 genes containing CGTCA motifs may mediate jasmonic acid signaling-mediated defense responses, while 17 ARE-containing genes potentially regulate anaerobic stress adaptation. Subcellular localization features indicate that nuclear-localized members (e.g., *BnaA03T0002600WE*) likely regulate downstream transcriptional networks under stress conditions through their ABRE/CGTCA-enriched promoters, whereas chloroplast-localized isoforms (e.g., *BnaA09T0052100WE*) imply TRM proteins may participate in photosynthesis or stress metabolic pathways by modulating cytoskeletal dynamics.

QRT-PCR validation further confirmed these predictions: *BnaA03T0002600WE* showed significant upregulation under drought and salt stress with vascular tissue-specific expression, suggesting its role in vascular system stress signaling transduction. Gene *BnaA02T0162800WE* exhibited consistently elevated expression under low-temperature, drought, and salt stresses, while *BnaA10T0057200WE* displayed downregulation across these conditions, indicating their divergent regulatory roles in plant abiotic stress adaptation. *BnaA02T0162800WE* may function as a positive regulator by modulating osmolyte accumulation, enhancing antioxidant defenses, or activating stress-responsive pathways to improve stress tolerance. Conversely, *BnaA10T0057200WE* potentially acts as a negative regulator, suppressing growth-related pathways to facilitate stress adaptation. Additionally, distinct *BnaTRM* genes exhibited early-response, late-response, or biphasic expression patterns, reflecting the functional diversity of *TRM* family members in plant multilevel stress adaptation mechanisms. Through targeted CRISPR-Cas9-mediated genome editing of genes, we aim to establish genotype–phenotype correlations by quantifying stress tolerance metrics (electrolyte leakage, ROS scavenging capacity) in engineered rapeseed lines and further investigation of their molecular mechanisms and functional validation. It will help elucidate their precise roles in rapeseed stress resistance and provide novel theoretical foundations for molecular breeding.

### 3.3. Limitations and Future Directions

While this study establishes foundational insights into the biological functions of *TRM* genes in polyploid plants, several critical questions warrant further investigation: First, dynamic subcellular studies employing live-cell imaging of GFP-tagged TRM proteins are required to delineate TRM-mediated microtubule reorganization during stress recovery phases. Second, protein interaction analyses through co-immunoprecipitation (Co-IP) experiments are necessary to validate TRM interactions with TON1/PP2A subunits in *Brassica napus*, coupled with systematic characterization of the TTP (TON1-TRM-PP2A) complex’s dynamic behavior and molecular mechanisms in microtubule remodeling. Third, epigenetic profiling should be conducted to identify histone modification signatures (e.g., H3K4me3/H3K27me3) at *TRM* gene loci. These advanced investigations will systemically integrate omics predictions with functional validation, thereby refining our understanding of *TRM* regulatory networks and providing precise molecular targets for crop genetic improvement. However, it is crucial to acknowledge that current findings remain confined to in silico predictions; subsequent functional characterization through CRISPR-based overexpression or knockout mutants will be essential to validate gene-specific roles in stress adaptation.

Future development of an integrated *BnaTRM* database combining proteomic and single-cell transcriptomic datasets will enable systems-level dissection of TRM-mediated signaling networks. Furthermore, integration with CRISPR-based genome editing technologies will facilitate precise plant functional characterization of *TRM* genes in stress responses. Identification of these key *TRM* genes provides directly applicable genetic targets for molecular breeding in *Brassica napus*, establishing foundational resources for developing stress-resilient varieties. For instance, constitutive promoter-driven overexpression of *BnaA02T0162800WE* can significantly enhance cold, drought, and salt tolerance in elite cultivars, generating transgenic plants with multistress resistance, while CRISPR-mediated knockout of *BnaA10T0057200WE* could release its negative regulatory effect, markedly improving plant stress tolerance. The translational application of these *TRM* genetic engineering strategies holds significant socioeconomic value: cultivation of *TRM*-modified varieties is projected to reduce global rapeseed yield losses caused by abiotic stresses, thereby enhancing agricultural sustainability and production stability.

## 4. Materials and Methods

### 4.1. Identification of the TRM Family

The BLASTp program (e < 1 × 10^−5^) was used to search for TRM candidate protein sequences (TAIR 10.1) in the whole-genome protein sequences of *Brassica napus* (Westar.v0) by using *Arabidopsis thaliana TRM* family proteins as bait sequences. Then, the candidate protein sequence of TRM in *Brassica napus* was analyzed, and the sequence containing *TRM* Motif was reserved as a member of the *TRM* family. Genes that do not conform to *TRM* family characteristics are removed and visualized by TBTOOLs v2.310 software. In this study, we used the following database to retrieve *TRM* sequences: TAIR database (TAIR10, https://www.arabidopsis.org/ (accessed on 5 March 2024)), Multiomics information resources of *Brassica napus* (BNIR) (https://yanglab.hzau.edu.cn/ (accessed on 5 March 2024)), National Center for Biotechnology Information (NCBI, www.ncbi.nlm.nih.gov (accessed on 1 April 2024)), MEME v5.5.7 (Multiple Expectation maximization for Motif Elicitation) (https://meme-suite.org/meme/tools/meme (accessed on 1 April 2024)).

### 4.2. Chromosome Location

We drew the chromosome map of *BnaTRM* family genes with TBTOOLs v2.310 software. The genome annotation file Westar.v0.GFF3 used in it comes from the website of BNIR (https://yanglab.hzau.edu.cn/ (accessed on 3 May 2024)).

### 4.3. Sequence Alignment and Phylogeny Analysis of BnaTRMs

We used MEGA 11.0.13 software to perform a phylogenetic analysis of the *TRM* families of *Brassica napus* and *Arabidopsis thaliana*. The built-in ClustalW program was used for multiple sequence alignments, and the neighbor-joining (NJ) method with a bootstrap value of 1000 repetitions was used to construct the phylogenetic tree. And the iTOL website (https://itol.embl.de/ (accessed on 25 May 2024)) was used to visualize the phylogenetic tree.

### 4.4. Synteny Analysis of BnaTRM Genes

To investigate the collinearity of *BnaTRM* genes with other plant species, we employed the One Step McScanX program in TBtools v2.310 to generate collinear files. Genome data of *Brassica napus* (Westar.v0), *Arabidopsis thaliana* (TAIR 10.1), soybean (*Glycine max* v2.1), rice (*Oryza sativa* L. 1.0), and maize (*Zea mays* L. 5.0) came from https://plants.ensembl.org/index.html (accessed on 14 June 2024). The results are visualized and built by TBtools v2.310.

### 4.5. The Biophysical Properties of BnaTRMs

The relative molecular weight (Mw), amino acid number (AA), and theoretical pI of protein, a member of the *TRM* family gene in *Brassica napus*, were calculated by ExPasy (https://www.expasy.org (accessed on 24 July 2024)). The subcellular localization of BnaTRM proteins was predicted with the WoLF PSORT online tool (https://wolfpsort.hgc.jp/ (accessed on 24 July 2024)).

### 4.6. The Identification of Cis-Regulatory Elements in Promoter Regions

To identify the putative cis-acting elements found in *BnaTRM* genes, the 2.0 kb genomic sequence upstream of the initiation codon (ATG) of each gene was subjected to PlantCARE (https://bioinformatics.psb.ugent.be/webtools/plantcare/html/ (accessed on 28 December 2024)). The results were visualized and constructed using TBtools v2.310.

### 4.7. RNA Isolation and Quantitative Real-Time PCR (qRT-PCR) Analysis

For gene expression analysis, 7-day-old wild-type Westar seedlings were grown in black plastic culture containers with Hoagland nutrient solution. After 14 days, the seedling roots were exposed to simulated drought stress with 20% PEG-6000 and salt stress with 200 mM NaCl solution. Four treatment time points were set: 0 h (control), 3 h, 12 h, and 48 h. Immediately after each treatment, leaf tissue samples were collected for subsequent RNA extraction. Total RNA was extracted using the Plant (Polysaccharide and Polyphenols) RNA Extraction Kit (Easy-DO, Hangzhou, China), and then we verified the quality and measured concentration. For cDNA Synthesis with the FastKing gDNA Dispelling RT SuperMix (TIANGEN, Beijing, China), qRT-PCR was performed using the UltraSYBR Mixture (Cwbio, Jiangsu, China) with CFX Opus 96 real-time PCR system (BIO-RAD, Hercules, CA, USA). The gene of *BnaC09T0180700WE* served as a reference gene. Three biological replicates were included in the expression analysis. The relative expression levels of *BnaTRMs* were calculated using the 2^−∆∆Ct^ approach. Primers are listed in Table S1.

## 5. Conclusions

This study presents a comprehensive investigation of the *TRM* gene family in *Brassica napus*, employing an integrative, multidisciplinary framework that combines bioinformatics, comparative genetics, and rigorous experimental validation to systematically explore the genomic architecture, functional diversity, and agronomic relevance of *BnaTRM* genes. Regarding the genomic organization, this study reveals that *BnaTRM* genes exhibit a distinct and uneven chromosomal distribution. Specifically, 87% of these genes are localized to the nucleus, while the remaining 13% are found in chloroplasts or cytoplasm. Furthermore, the genes cluster on chromosomes A09 or C09, suggesting a potential hot spot for *TRM* gene activity. The extensive synteny observed between *BnaTRM* genes and those in soybeans underscores the evolutionary conservation of this gene family across species.

The functional diversity of *BnaTRM* genes is also illuminated in this study. By analyzing promoter-enriched cis-elements, such as ABRE and ARE, we discovered that these elements play a crucial role in regulating gene expression. Additionally, the biphasic expression patterns observed under stress conditions implicate *BnaTRM* genes in hormone signaling and abiotic stress adaptation. This finding suggests that these genes may play a pivotal role in the plant’s response to environmental stressors. Through integrative analysis of the *BnaTRM* gene family, this study identified six stress-responsive candidate genes (*BnaA02T0162800WE*, *BnaA10T0057200WE*, *BnaA03T0002600WE*, *BnaA05T0045700WE*, *BnaC04T0062800WE*, and *BnaA02T0207500WE*) as key regulators of abiotic stress adaptation. Based on the results of this paper and previous studies [11,24,29,30], the present study proposes that *BnaTRMs* form synergistic regulatory modules by interacting with other stress-related proteins (such as *MYB* family transcription factors, ion transporters, and other related proteins) and are assembled into multisubunit complexes within plant cells. These complexes may dynamically regulate the expression of key genes (e.g., the *MYB* transcription factor family) in cold, drought, and salt stress response networks, thereby enhancing plant adaptability to multiple environmental stresses.

Collectively, this study advances our understanding of microtubule-mediated stress responses in polyploid crops and establishes a solid foundation for precision breeding strategies aimed at enhancing agricultural sustainability. The insights gained from this research have the potential to revolutionize crop breeding and improve the resilience of *Brassica napus* and other polyploid crops to environmental stressors, ultimately contributing to global food security and sustainability.

## Figures and Tables

**Table 1:** Information regarding the *BnaTRMs*.

	Gene ID	Amino Acid	Molecular Weight	pI	Instability Index	Aliphatic Index	GRAVY	Subcellular Location *
1	*BnaA01T0003500WE*	893	99,267.92	5.54	65.68	70.36	−0.673	nucl
2	*BnaA03T0020300WE*	442	50,428.59	5.46	58.99	76.45	−0.658	nucl
3	*BnaA02T0001100WE*	427	47,976.39	5.27	62.08	61.83	−0.937	chlo
4	*BnaA02T0005200WE*	427	47,976.39	5.27	62.08	61.83	−0.937	chlo
5	*BnaA02T0059900WE*	882	98,631.55	9.3	65.97	68.28	−0.851	nucl
6	*BnaA02T0162800WE*	771	85,854.29	9.15	56.42	72.68	−0.579	nucl
7	*BnaA02T0207500WE*	1004	111,547.12	9.27	63.28	71.27	−0.765	nucl
8	*BnaA02T0247600WE*	492	55,305.13	4.86	63.96	71.34	−0.61	nucl
9	*BnaA02T0371400WE*	682	75,521.09	9.09	55.4	68.3	−0.681	nucl
10	*BnaA03T0002600WE*	274	31,918.15	4.37	72.97	72.15	−0.777	nucl
11	*BnaA03T0015300WE*	778	88,777.39	6.04	64.22	68.02	−0.921	nucl
12	*BnaA03T0189100WE*	399	45,643.76	4.96	63.38	64.81	−0.865	nucl
13	*BnaA03T0206700WE*	461	52,564.18	5.62	53.8	72.26	−0.775	nucl
14	*BnaA03T0290500WE*	798	90,854.57	5.49	57.45	80.88	−0.644	nucl
15	*BnaA03T0296800WE*	860	96,532.39	9.42	69.02	69.51	−0.786	nucl
16	*BnaA04T0010200WE*	647	72,419.75	4.84	64.08	80.7	−0.475	nucl
17	*BnaA04T0050800WE*	899	101,494.36	6.66	64.76	62.58	−0.938	cyto
18	*BnaA04T0223200WE*	413	47,155.49	5.27	69.42	64.72	−0.856	nucl
19	*BnaA04T0276400WE*	652	73,052.54	4.9	60.22	74.42	−0.657	nucl
20	*BnaA05T0045700WE*	674	76,122.5	4.78	58.66	66.53	−0.795	nucl
21	*BnaA05T0068200WE*	712	81,103.55	6.4	63.77	63.72	−0.904	nucl
22	*BnaA05T0075500WE*	436	49,818.09	5.07	63.45	59.27	−1.014	nucl
23	*BnaA06T0268400WE*	705	78,784.51	9.55	67.86	68.17	−0.823	nucl
24	*BnaA06T0467500WE*	198	22,545.77	4.76	50.63	97.42	−0.332	nucl
25	*BnaA07T0005300WE*	760	85,531.42	8.97	62.59	69.47	−0.697	nucl
26	*BnaA07T0028400WE*	766	87,786.1	5.69	66.14	69.18	−0.841	nucl
27	*BnaA07T0061900WE*	722	80,996.43	9.62	62.23	69.92	−0.864	nucl
28	*BnaA07T0178000WE*	854	96,069.8	7.18	68.97	64.18	−0.905	nucl
29	*BnaA07T0198700WE*	800	88,413.82	8.21	54.93	67.96	−0.698	nucl
30	*BnaA07T0284900WE*	806	89,215.04	9.2	51.71	72.72	−0.591	nucl
31	*BnaA08T0155700WE*	445	50,683.9	9.84	67.8	64.61	−0.969	nucl
32	*BnaA08T0233600WE*	828	92,656.53	9.1	61.85	74.86	−0.738	nucl
33	*BnaA08T0282300WE*	508	59,099.71	6.16	53.22	77.28	−0.828	nucl
34	*BnaA09T0005200WE*	860	97,167.81	5.86	57.61	82.55	−0.6	chlo
35	*BnaA09T0052100WE*	814	90,343.02	5.89	55.09	69.07	−0.666	nucl
36	*BnaA09T0069100WE*	447	49,538.56	9.86	65.45	65.01	−0.851	nucl
37	*BnaA09T0138200WE*	511	59,130.95	6.02	52.86	85.4	−0.72	nucl
38	*BnaA09T0176000WE*	815	92,650.45	9.01	44.57	71.15	−0.929	nucl
39	*BnaA09T0182600WE*	755	84,694.03	5.45	61.55	75.79	−0.652	chlo
40	*BnaA09T0234700WE*	476	53,576.42	4.72	60.88	73.09	−0.571	nucl
41	*BnaA09T0466000WE*	856	95,817.42	6.58	65.54	66.07	−0.837	nucl
42	*BnaA09T0523800WE*	642	72,259.34	4.82	57.86	81.18	−0.579	nucl
43	*BnaA09T0538400WE*	544	61,533.42	9.12	80.14	77.41	−0.664	nucl
44	*BnaA09T0580700WE*	835	93,263.41	9.61	66.2	73.53	−0.756	nucl
45	*BnaA09T0662300WE*	460	52,907.51	5.7	55.41	72.67	−0.746	nucl
46	*BnaA10T0057200WE*	539	61,801.35	6.47	52.29	70.13	−0.847	nucl
47	*BnaA10T0086600WE*	493	55,727.57	9.82	70.03	71.18	−0.886	nucl
48	*BnaA10T0204100WE*	896	99,567.65	9.29	69.04	64.98	−0.814	nucl
49	*BnaA10T0283700WE*	506	57,832.3	5.04	71.35	66.44	−0.782	nucl
50	*BnaA10T0290700WE*	788	89,566.8	5.21	69.87	71.12	−0.899	nucl
51	*BnaC01T0073000WE*	867	96,464.1	5.89	65.2	69.33	−0.683	nucl
52	*BnaC02T0063200WE*	852	95,550.24	9.31	65.89	67.27	−0.83	nucl
53	*BnaC02T0201300WE*	751	83,971.47	9.08	58.09	75.17	−0.585	nucl
54	*BnaC02T0270900WE*	998	111,051.48	9.3	61.59	69.66	−0.797	nucl
55	*BnaC02T0346900WE*	469	52,939.49	4.96	61.14	70.04	−0.674	nucl
56	*BnaC02T0484200WE*	710	79,307.97	9.03	58.19	69.86	−0.684	nucl
57	*BnaC03T0141700WE*	402	46,007.15	4.94	64.13	64.58	−0.879	nucl
58	*BnaC03T0163900WE*	464	52,831.39	5.6	58.24	72.82	−0.766	nucl
59	*BnaC03T0270700WE*	750	85,781.03	5.65	53.28	81.37	−0.677	nucl
60	*BnaC03T0277600WE*	864	97,194.43	9.54	67.49	69.64	−0.794	nucl
61	*BnaC03T0482600WE*	716	80,290.29	9.5	66.4	68.76	−0.832	nucl
62	*BnaC04T0062800WE*	698	78,849.91	4.94	60.02	66.48	−0.778	nucl
63	*BnaC04T0093600WE*	726	82,317.98	6.22	63.06	65.72	−0.825	nucl
64	*BnaC04T0102900WE*	404	46,423.23	4.94	68.45	60.59	−1.045	nucl
65	*BnaC04T0261700WE*	673	75,663.01	4.7	63.19	79.29	−0.582	nucl
66	*BnaC04T0281700WE*	682	75,880.55	8.27	51.54	69.87	−0.645	nucl
67	*BnaC04T0322500WE*	889	99,862.63	6.33	67.2	64.38	−0.871	cyto
68	*BnaC04T0535300WE*	416	47,449.8	5.41	65.58	63.1	−0.891	nucl
69	*BnaC04T0593100WE*	657	73,552.03	4.99	62.64	73.87	−0.669	nucl
70	*BnaC05T0054600WE*	539	62,144	6.15	56.71	78.63	−0.756	nucl
71	*BnaC05T0554900WE*	833	93,695.01	9.57	60.21	66.37	−0.866	nucl
72	*BnaC05T0557900WE*	823	92,629.67	9.33	61.49	66.34	−0.841	nucl
73	*BnaC06T0172800WE*	898	100,912.08	6.38	67.1	65.49	−0.874	nucl
74	*BnaC06T0241500WE*	807	89,398.11	8.46	55.86	67.01	−0.711	nucl
75	*BnaC06T0356200WE*	792	87,809.97	9.12	53.76	71.91	−0.632	nucl
76	*BnaC07T0002300WE*	716	80,145.54	6.56	64.23	68.45	−0.688	nucl
77	*BnaC07T0043500WE*	771	88,206.85	5.93	62.67	71.62	−0.788	nucl
78	*BnaC07T0077300WE*	727	81,314.9	9.61	62.42	70.25	−0.837	nucl
79	*BnaC07T0178800WE*	814	92,518.49	9.1	43.29	72.09	−0.915	nucl
80	*BnaC07T0185500WE*	750	84,253.74	5.62	62.17	76.67	−0.636	chlo
81	*BnaC07T0220600WE*	812	91,124.51	5.43	62.07	75.12	−0.594	chlo
82	*BnaC08T0003900WE*	512	59,406.12	5.93	51.39	78.96	−0.817	nucl
83	*BnaC08T0203100WE*	821	92,480.81	9.48	60.94	73.7	−0.791	nucl
84	*BnaC08T0287100WE*	850	95,141.58	6.56	66.64	65.06	−0.85	nucl
85	*BnaC08T0351700WE*	657	73,800.16	4.85	60.68	80.38	−0.571	nucl
86	*BnaC08T0368900WE*	550	61,796.54	8.97	78.06	76.42	−0.655	nucl
87	*BnaC08T0416900WE*	845	94,589.65	9.43	68.48	74.6	−0.762	nucl
88	*BnaC09T0001400WE*	823	93,463.15	5.69	60.18	77.98	−0.713	nucl
89	*BnaC09T0051500WE*	817	90,780.52	6.3	56.05	69.41	−0.681	nucl
90	*BnaC09T0078200WE*	448	49,796.94	9.86	62.41	66.83	−0.81	nucl
91	*BnaC09T0148800WE*	640	73,819.33	7.17	61.94	75.47	−0.847	nucl
92	*BnaC09T0166000WE*	525	60,759.83	5.68	52.79	83.47	−0.735	nucl
93	*BnaC09T0268000WE*	472	53,039.08	4.85	62.94	76.99	−0.508	nucl
94	*BnaC09T0317500WE*	492	55,333.7	9.8	67.92	68.74	−0.861	nucl
95	*BnaC09T0394800WE*	347	39,940.55	5.9	64.51	68.21	−0.945	nucl
96	*BnaC09T0480100WE*	902	100,381.76	9.18	69.3	68.4	−0.766	nucl
97	*BnaC09T0579200WE*	627	70,490.47	5.33	71.22	64.51	−0.72	nucl
98	*BnaC09T0587700WE*	849	96,506.85	5.32	66.98	71.74	−0.864	nucl
99	*Bnascaffold2730T0005700WE*	778	88,777.39	6.04	64.22	68.02	−0.921	nucl
100	*Bnascaffold3320T0000100WE*	838	95,324.53	5.27	66.12	72.1	−0.866	nucl

* nucl, chlo, and cyto indicate nucleus, chloroplast, and cytoplasm.

**Table 2:** Number of hormone and stress-responsive cis-elements in the promoters of the *BnaTRMs*.

	Cis-Elements	Function of Cis-Elements	Number of Genes
1	3-AF1 binding site	light-responsive element	6
2	AACA_motif	involved in endosperm-specific negative expression	2
3	ABRE	cis-acting element involved in the abscisic acid responsiveness	17
4	ACE	cis-acting element involved in light responsiveness	3
5	AE-box	part of a module for light response	9
6	ARE	cis-acting regulatory element essential for the anaerobic induction	17
7	ATC-motif	part of a conserved DNA module involved in light responsiveness	2
8	ATCT-motif	part of a conserved DNA module involved in light responsiveness	4
9	AT-rich element	binding site of AT-rich DNA binding protein (ATBP-1)	7
10	AuxRR-core	cis-acting regulatory element involved in auxin responsiveness	1
11	Box 4	part of a conserved DNA module involved in light responsiveness	18
12	Box II	part of a light-responsive element	2
13	CAG-motif	part of a light-response element	1
14	CAT-box	cis-acting regulatory element related to meristem expression	3
15	CCAAT-box	MYBHv1 binding site	4
16	CGTCA-motif	cis-acting regulatory element involved in the MeJA responsiveness	16
17	chs-CMA1a	part of a light-responsive element	3
18	chs-CMA2a	part of a light-responsive element	1
19	circadian	cis-acting regulatory element involved in circadian control	4
20	GA-motif	part of a light-responsive element	3
21	GARE-motif	gibberellin-responsive element	9
22	GATA-motif	part of a light-responsive element	4
23	G-Box	cis-acting regulatory element involved in light responsiveness	17
24	GCN4_motif	cis-regulatory element involved in endosperm expression	2
25	GT1-motif	Light-responsive element	10
26	GTGGC-motif	part of a light-responsive element	1
27	I-box	part of a light-responsive element	9
28	LAMP-element	part of a light-responsive element	3
29	LS7	part of a light-responsive element	1
30	LTR	cis-acting element involved in low-temperature responsiveness	6
31	MBS	MYB binding site involved in drought-inducibility	10
32	MBSI	MYB binding site involved in flavonoid biosynthetic gene regulation	5
33	MRE	MYB binding site involved in light responsiveness	6
34	O2-site	cis-acting regulatory element involved in zein metabolism regulation	6
35	P-box	gibberellin-responsive element	14
36	RY-element	cis-acting regulatory element involved in seed-specific regulation	3
37	Sp1	light-responsive element	3
38	TATC-box	cis-acting element involved in gibberellin responsiveness	3
39	TCA-element	cis-acting element involved in salicylic acid responsiveness	10
40	TCCC-motif	part of a light-responsive element	3
41	TC-rich repeats	cis-acting element involved in defense and stress responsiveness	15
42	TCT-motif	part of a light-responsive element	28
43	TGA-box	part of an auxin-responsive element	1
44	TGACG-motif	cis-acting regulatory element involved in the MeJA responsiveness	16
45	TGA-element	auxin-responsive element	10
46	WUN-motif	wound-responsive element	1

## Data Availability

The data presented in this study are available in the paper and Supplementary Files.

## Supplementary Materials

The following supporting information can be downloaded at: https://www.mdpi.com/article/10.3390/plants14121858/s1, Table S1: Primer sequences used for RT-qPCR.
